# Drug Combination in Polymeric Nanocarriers for Chemotherapy of Cancer: Preclinical Outcomes in the Last Ten Years

**DOI:** 10.3390/ph19020248

**Published:** 2026-02-01

**Authors:** Fernanda Karoline Vieira da Silva Torchelsen, Eduardo Burgarelli Lages, Maria Alice de Oliveira, André Luís Branco de Barros, Vanessa Carla Furtado Mosqueira

**Affiliations:** 1 Núcleo de Pesquisa em Ciências Biológicas, Universidade Federal de Ouro Preto, Ouro Preto 35400-000, Minas Gerais, Brazil; 2 Departamento de Produtos Farmacêuticos, Universidade Federal de Minas Gerais, Belo Horizonte 31270-901, Minas Gerais, Brazil; eduardoburgarelli@ufmg.br; 3 Escola de Farmácia, Universidade Federal de Ouro Preto, Ouro Preto 35400-000, Minas Gerais, Brazil; maria.alice@ufop.edu.br (M.A.d.O.);; 4 Departamento de Análises Clínicas e Toxicológicas, Universidade Federal de Minas Gerais, Belo Horizonte 31270-901, Minas Gerais, Brazil; brancodebarros@yahoo.com.br

**Keywords:** polymeric nanocarriers, drug co-encapsulation, tumor growth inhibition, synergistic efficacy, preclinical study

## Abstract

**Background:** Combination chemotherapy using nanotechnology-based delivery is a promising approach to improve cancer treatment, but the added value of co-loaded polymeric nanocarriers has not been comprehensively appraised. This review synthesizes preclinical evidence on polymeric systems co-encapsulating antitumor agents. **Methods:** A narrative literature review identified 33 preclinical studies (2015–2025) employing polymer-based nanocarriers to co-load at least two antitumor drugs. Study characteristics and in vitro and in vivo outcomes were qualitatively analyzed. **Results:** Most studies addressed breast, lung, or ovarian cancer and used micelles or nanospheres. Co-loaded formulations consistently enhanced in vitro cytotoxicity and, in vivo, produced marked tumor growth inhibition relative to free drugs or single-loaded systems; in several reports, near-complete or complete tumor regression was achieved. Synergy was frequently suggested but not consistently quantified, more than half of the studies did not report a combination index. Most formulations showed favorable tolerability, with few reports including mild hepatic toxicity, renal, or weight-related effects. Beyond conventional drug pairs, examples of co-delivering chemotherapeutics with resistance modulators, gene therapy agents, or targeted ligands illustrated how tailored release profiles and active targeting can potentiate efficacy. Nevertheless, heterogeneity in models, dosing schedules, endpoints, and limited long-term safety data hamper cross-study comparison and translation. **Conclusions:** Co-loaded polymeric nanocarriers constitute a promising platform to optimize combination chemotherapy, improving preclinical antitumor efficacy with generally limited toxicity, but more standardized and mechanistically driven studies are required to support clinical development.

## 1. Introduction

Despite remarkable advances in medicine over recent decades, particularly regarding early diagnosis and the development of innovative therapeutic modalities, cancer remains one of the leading causes of death worldwide. According to the International Agency for Research on Cancer (IARC) of the World Health Organization (WHO), approximately 20 million new cases and 9.7 million deaths from cancer were reported in 2022 [1]. Moreover, IARC’s Global Cancer Observatory projections suggest a substantial increase in both cancer incidence and prevalence in the coming decades, with around 35 million new cases expected by 2050, representing a 75% increase compared to 2022 [2].

Among the various cancer types, lung cancer shows the highest incidence worldwide (2.48 million cases in 2022; 12.4%), followed by breast cancer (2.3 million cases; 11.5%), colorectal cancer (1.9 million cases; 9.6%), and prostate cancer (1.5 million cases; 7.3%) [1]. Given these data and the increasing burden of cancer worldwide, the development of new, more effective and efficient therapeutic strategies remains a challenge for science and the subject of numerous studies.

The choice of oncological treatment depends on several factors, including tumor type, disease stage, and the individual characteristics of each patient. In general, the main therapeutic modalities comprise surgery, radiotherapy, and chemotherapy. However, the limited specificity of conventional antitumoral drugs remains a major limitation, frequently associated with severe adverse effects and systemic toxicities [3]. The complexity and heterogeneity of tumor biology intensify the therapeutic challenges. Cancer cells often exhibit genetic instability, dysregulated signaling pathways, and adaptive mechanisms that enable survival under pharmacological treatment. The intrinsic or acquired resistance of tumor cells to standard chemotherapeutic drugs represents a considerable obstacle to successful cancer therapy [4,5]. 

These characteristics highlight the need for therapeutic approaches that improve the efficacy and selectivity of antitumor treatments, especially those capable of acting on multiple molecular targets simultaneously, and consequently reducing side effects. Among these, combined chemotherapy and nanotechnology-based drug delivery systems have emerged as two of the most promising approaches to overcome the limitations of conventional treatments.

Combination chemotherapy involves the simultaneous administration of two or more drugs or active compounds with different mechanisms of action, aiming for an additive or synergistic effect. By acting on multiple targets, combination treatments can inhibit tumor proliferation, angiogenesis, and metastasis more effectively than single-drug treatments. Furthermore, this approach can delay or prevent the development of drug resistance by limiting the tumor’s ability to adapt to a single pharmacological stimulus. Synergistic drug combinations also allow for lower individual doses, thus reducing systemic toxicity and improving patient tolerability [6,7,8].

In parallel, advances in nanotechnology-based drug delivery systems have provided a means of increasing the selectivity and bioavailability of chemotherapeutic agents. Since the U.S. Food and Drug Administration (FDA) approval of Doxil^®^, the first nanomedicine formulation consisting of liposomal doxorubicin, in 1995, the incorporation of active compounds into nanostructured delivery systems has been extensively investigated. The encapsulation of chemotherapeutic agents within nanocarriers, such as liposomes [9,10,11], polymeric nanoparticles [12,13], and micelles [14,15,16], offers several advantages, including improved drug dispersity, protection from premature degradation, enhanced tumor targeting, controlled release, and reduced systemic toxicity.

Although lipid-based systems like liposomes are clinically established, they often face limitations regarding instability and premature drug leakage. Polymeric nanocarriers address these challenges, offering distinct advantages for complex co-delivery strategies. Unlike their lipidic counterparts, polymeric nanoparticles exhibit high structural stability in biological fluids and allow for precise control over release kinetics. Furthermore, the chemical versatility of polymers enables sophisticated surface functionalization and the loading of diverse therapeutic agents, significantly expanding the scope of treatable conditions [17].

**Figure 1:**
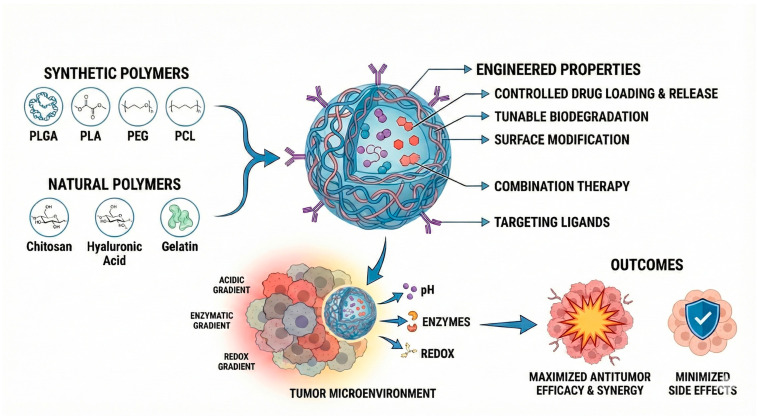
Engineered polymeric systems for anti-tumoral activity and the main constituents and approaches to improve selectivity. Abbreviations: PLGA, Poly(lactic-co-glycolic acid); PLA, Polylactic acid; PEG, polyethylene glycol; PCL, Polycaprolactone.

Building on these advantages, among the wide range of nanostructured platforms explored for the development of innovative cancer therapies, polymeric systems are promising due to their ability to be rationally engineered with tunable physicochemical and functional properties, which are strongly dictated by the polymer composition and architecture. Polymeric nanoparticles (Figure 1), whether based on biodegradable synthetic polymers (such as poly(lactic-co-glycolic acid), polylactide, and polycaprolactone) or natural polymers (such as chitosan, hyaluronic acid, and collagen) and their copolymers with polyethylene glycol allow for control over drug loading, release kinetics, biodegradation rate, and surface modification. Additionally, these systems enable the incorporation of targeting ligands [18,19,20,21,22] and the construction of stimuli-responsive architectures capable of releasing drugs in response to pH [18,23,24,25], enzymatic activity [26,27], or redox gradients [21,28] within the tumor microenvironment. Due to these attributes, polymeric nanocarriers have become one of the most widely studied approaches for next-generation cancer therapy.

Considering the benefits observed with these two strategies, the integration of nanotechnology with drug combination has shown particularly promising results for the development of new cancer treatments. The simultaneous encapsulation of multiple drugs in a single nanocarrier allows for the synchronized administration of active ingredients, maintaining the ideal proportions of each drug at the tumor site and maximizing therapeutic synergy [29,30]. This approach not only enables increased antitumor efficacy but also minimizes side effects [23,31,32,33], representing an important strategy to be explored in the development of new cancer treatments.

While the physicochemical design of nanocarriers has been extensively discussed in the literature, questions remain regarding the extent to which these systems effectively translate into meaningful therapeutic benefits. In this review, we focus on polymeric co-delivery platforms and examine whether co-encapsulation strategies consistently improve antitumor outcomes in preclinical models. By analyzing studies published over the last decade (2015–2025), we highlight recurring challenges related to synergy assessment, in vivo experimental consistency, and safety evaluation. Finally, we propose an integrative perspective that links nanocarrier design choices to biological mechanisms, with the aim of supporting a more rational interpretation and development of polymeric co-delivery systems.

## 2. Mapping of Cancer Types and Nanocarrier Co-Delivery Studies

An analysis of 33 studies from the last ten years concerning combined and co-loaded drugs in nanoparticle systems revealed diverse strategies for drug entrapment, and frequent use of modified polymers. To streamline the evaluation of therapeutic efficacy, this section will detail the main outcomes reported in both in vitro and in vivo studies. The primary endpoints considered are the in vitro effects and the in vivo anti-tumor activity of the combined therapies.

A quantitative analysis of the literature reviewed reveals that micelles were the predominant nanocarrier system for co-loaded drugs (45.45%), followed by nanospheres (36.36%). While all studies investigated drug combinations, 51.51% did not calculate the Combination Index (CI), a key metric for determining synergy. Despite this, the therapeutic outcomes were consistently favorable. All studies reported enhanced in vitro effects, and in vivo experiments demonstrated significant tumor suppression relative to control treatments. Meanwhile, a lack of standardization in experimental design and reporting hindered meaningful comparisons between study results. The research varied widely in terms of the cell lines, animal tumor models, and treatment schedules used. In particular, there was no consensus for reporting in vivo results, with endpoints cited as tumor volume, growth rate, or final weight, compromising a unified analysis.

The results of the reviewed articles are detailed below (Table 1), categorized by the type of targeted tumor cells. 

### 2.1. Breast Cancer

Most articles found were focused on breast cancer (39.39%), 46.15% of the works used doxorubicin combined with another drug, and 30.76% combined paclitaxel with a different drug. The selection of these cornerstone chemotherapeutic agents suggests a primary research goal of enhancing current clinical treatment regimens. Among the nanocarrier systems, the most used were micelles (53.84%), followed by nanospheres (30.76%), and nanocapsules or core-shell nanoparticles (NP) (7.69%). A significant methodological omission was noted, as 61.54% of the studies did not report the CI, a critical metric for confirming synergistic drug interactions.

A lack of methodological consensus was apparent in the in vitro assessments, which utilized diverse cell lines (e.g., 4T1, MCF7, MCF/ADR, MDA-MB-231) and a wide range of functional assays, including those for cell proliferation, migration, and cell cycle analysis. Similarly, in vivo experiments were conducted in various tumor-bearing mouse models, such as those with Ehrlich ascites carcinoma, MCF-7/ADR, or 4T1 tumors.

Treatment protocols also vary in frequency or dosage. The shortest regimen identified was docetaxel plus silibinin micelle treatment two times for two weeks (2x/2 w) [42], and the highest frequency of treatment was seen with doxorubicin and salinomycin 5 mg/kg every two days for sixteen days (2 d/16 d) [41]. Intravenous (IV) injection was the most predominant route of administration (92.85%), with one exception of intraperitoneal delivery [46] (Behl et al., 2023).

Regarding therapeutic efficacy, 50% of the studies provided quantitative data for tumor suppression. From these, the most pronounced result was 97% of tumor reduction [40] with nanospheres of salinomycin (1 mg/kg) and doxorubicin (3 mg/kg) administered twice a week for 3 weeks. Other notable findings included ongoing tumor inhibition effects even after drug withdrawal and metastasis prevention [41], using a different combination of doxorubicin:salinomycin 5 mg/kg, given every two days for 16 days. 

Most of the studies did not report significant toxicity. One exception noted a slight increase in alanine aminotransferase (ALT) and aspartate aminotransferase (AST) levels when docetaxel (2 mg/kg) and thymoquine (4 mg/kg) were administered every three days for four cycles [36].

### 2.2. Lung Tumors

Lung cancer was the second most frequently investigated malignancy, comprising 27.3% of the reviewed articles. In vitro studies predominantly used tumoral lung lineage A549 and H460, but also including 344SQ, NCL-H460, HUVECs, H69AR, LLC, or SKOV-3 cells. In vivo research most employed mouse models bearing tumors derived from Lewis Lung Carcinoma (LLC), A549, or H460 cells. All studies successfully demonstrated in vitro efficacy, typically by assessing the inhibition of cell proliferation or enhanced cytotoxicity.

The most common therapeutic agent investigated for co-delivery was paclitaxel (5/9 articles), followed by cisplatin (4/9), etoposide (2/9), and doxorubicin (2/9). Nanospheres were the predominant drug delivery system (55.55%). Notably, in contrast to the breast cancer literature, most of these studies (66.7%) reported the CI, quantitatively confirming synergistic activity between the co-administered agents.

In vivo dosing regimens were highly variable, even for the same therapeutic agent; for instance, paclitaxel was administered at doses ranging from 1 mg/kg to 7.5 mg/kg. Treatment durations also varied significantly. The shortest protocol consisted of a single administration of nanospheres of etoposide (1.25 mg/kg) and cisplatin (2.25 mg/kg) [32] while the longest involved seven cycles of nanospheres containing paclitaxel (1 mg/kg) and carboplatin (2 mg/kg) [54].

Most studies did not report tumor reduction as a percentage; instead, they compared the efficacy of the combination therapy to control groups (free drug, NPs of the isolated drug). Among the works that provide quantitative data, one [55] reported 83% of tumor inhibition, although its posology (micelles of doxorubicin combined with paclitaxel) was unclear in the article. Another work achieved almost complete suppression of the tumor (although not presented percentage) with the administration of micelles containing doxorubicin (5 mg/kg) and irinotecan (50 mg/kg), along with three administrations [52].

### 2.3. Ovarian Tumor

Four of the reviewed articles focused on ovarian cancer, investigating the co-loading of drugs in polymeric nanoparticles. The articles vary on drug combinations used, typically maintaining a conventional chemotherapeutic agent and combining it with an experimental drug. The following combinations were: adavosertib + alaparib, doxorubicin + verapamil, rhein + doxorubicin, wortmannin + cisplatin. Nanostructures used for delivery include micelles, nanocapsules, and nanospheres. Two [31,60] of the four studies quantitatively confirmed synergistic interactions by calculating the CI from their in vitro assays. 

The in vivo tumor assessment showed that all NPs could diminish tumor size. The most significant efficacy was a 93.5% tumor reduction, achieved with nanocapsules co-delivering adavosertib (5 mg/kg) and olaparib (25 mg/kg) administered every two days for six cycles [60].

### 2.4. Liver Tumors

Research on liver cancer accounted for 4 of the 33 reviewed articles. The in vitro studies utilized hepatocarcinoma cell lines (HCC), SMMC 7721, Huh-7, and LX-2. The in vivo experiments were conducted in a mouse model with HCC, SMMC 7721, or H22/m-HSC tumors.

Doxorubicin was the most frequently employed therapeutic, appearing in 75% of the works in combination with another drug or active, delivered in nanospheres or micelles. Notably, plumbagin, a naphthoquinone with in vitro and in vivo anticancer activities, and immunogenic cell death potential [50] occurred in 50% of the co-loaded NP. Half of the studies reported CI. The in vitro assays evaluated migration (50%) [49,51] and proliferation (50%) [30,50].

All co-loaded formulations demonstrated in vivo tumor suppression. The most significant efficacy was an 85.5% of tumor growth inhibition, achieved with micelles co-delivering doxorubicin (3 mg/kg) and all-trans retinoic acid (2.2 mg/kg), every two days for seven cycles [51]. This treatment regimen also decreased lung metastasis.

### 2.5. Other Cancers

Research on other malignancies was limited, with only a single article identified for each of the following: cervical, colon, and lymphoma cancer.

Cervical cancer study [47] combined shRNA beclin 1 to doxorubicin in micelles, which improved endocytosis and tumor inhibition.Colon cancer study [48] used micelles containing paclitaxel prodrug combined with combretastatin A4, a compound that prevents microtubule polymerization leading to mitosis arrest. The treatment achieved 87.2% of tumor inhibition. A lymphoma study [59] was based on lipid polymeric NPs with vincristine and quercetin, a bioactive flavonoid known to reduce inflammation and oxidative stress. The treatment led to significant tumor growth inhibition.

## 3. Polymeric Nanocarrier Design Strategies for Drug Co-Delivery

Polymeric nanocarriers play an active and enabling role in the co-loading of multiple therapeutic agents, rather than merely serving as passive delivery vehicles. Their chemical composition, architecture, and structural organization allow the simultaneous incorporation of drugs with distinct physicochemical properties, including differences in solubility, molecular weight, and lipophilicity [55,62]. Structural features such as core-shell architectures, hydrophobic domains, and functionalized surfaces enable the spatial compartmentalization of co-encapsulated agents, thereby minimizing direct drug–drug incompatibilities and preserving individual drug activity [18,23,35,38]. This intrinsic structural versatility makes polymeric nanocarriers particularly suitable for co-loading strategies, as it enables the rational design of combination therapies within a single delivery system.

Co-loading multiple therapeutic agents into a single nanocarrier system offers several advantages over the administration of free drug combinations or separate carrier systems. By encapsulating multiple drugs within the same carrier, synchronized pharmacokinetics and biodistribution can be achieved, ensuring that the combined agents reach the target site at defined and coordinated ratios [40]. This spatial and temporal co-delivery is particularly important for maintaining pharmacological synergy and preventing suboptimal exposure of individual drugs. In addition, co-loading within a single nanocarrier can reduce systemic toxicity by limiting off-target exposure and enabling lower effective doses [30,36,60]. Importantly, this strategy may also help overcome drug resistance by simultaneously modulating complementary pathways within tumor cells and the tumor microenvironment, thereby enhancing therapeutic efficacy [33,34].

### 3.1. Co-Encapsulation of Conventional Chemotherapeutics

The combination of chemotherapeutic agents with distinct mechanisms of action represents a keystone strategy in oncology to enhance therapeutic efficacy, overcome dose-limiting toxicities, and combat multidrug resistance (MDR). Classic drug pairs, such as doxorubicin with paclitaxel or docetaxel with cisplatin, are widely used in clinics for various cancers. However, their co-administration in conventional formulations is often hampered by divergent pharmacokinetics, poor solubility, and non-selective biodistribution, which can lead to severe side effects and suboptimal tumor drug concentrations. In contrast, co-encapsulation confers coordinated pharmacokinetics, improved tumor exposure at predefined drug ratios, and the capacity to modulate release sequences that may enhance synergy while mitigating systemic toxicity [38,63]. 

A representative example is the PLA-based hybrid block-copolymer system developed by Anees et al. [40], which co-encapsulated salinomycin and doxorubicin at a 1:3 molar ratio. Salinomycin is a small-molecule ionophore with activity against cancer stem cells, whereas doxorubicin is a widely used cytotoxic agent targeting proliferating tumor cells. The authors showed that the PLA-based nanoparticles achieved high encapsulation efficiencies (>80%) and sustained release for both drugs, in addition to improving the pharmacokinetic and biodistribution profile of salinomycin, thereby reducing its off-target toxicity. The co-delivery system achieved a strong synergistic effect against several cancer cell lines, including MDA-MB 231, MDA-MB 468, SUM-149 and EAC, with CI values ranging from 0.17 to 0.58. In an Ehrlich ascites carcinoma model, the co-encapsulated nanoparticles drastically reduced the severe toxicity of salinomycin, allowing for a threefold higher maximum tolerated dose. Moreover, treatment with salinomycin:doxorubicin co-loaded nanoparticles resulted in near-complete tumor regression (~97% reduction) and prevented tumor recurrence, showcasing a promising strategy for durable cancer treatment.

In another example, Chen et al. [43] used pluronic-based functional micelles to co-deliver doxorubicin and paclitaxel, specifically addressing MDR. The authors synthesized a pluronic P105–doxorubicin conjugate that served as a hydrophobic core to entrap another anticancer drug, paclitaxel, with pluronic F127 to form the dual drug-loaded mixed micelles (PF–DP). Given that pluronics are known to modulate membrane efflux processes and interfere with P-gp activity, this mixed micelle architecture could additionally enhance drug retention and intracellular accumulation in resistant cells. The micelles exhibited favorable physicochemical properties, such as a particle size close to 22 nm and encapsulation efficiencies greater than 90% for both drugs. The formulation also improved in vitro cytotoxicity, drug uptake, and apoptosis induction in MDR breast cancer cells (MCF-7/ADR) compared to single-drug micelles or free drug combinations. These promising in vitro results were confirmed in vivo, where PF–DP micelles demonstrated superior inhibition of tumor growth in an MCF-7/ADR tumor-bearing mouse model and a more favorable safety profile, notably reducing cardiotoxicity associated with free doxorubicin.

**Figure 2:**
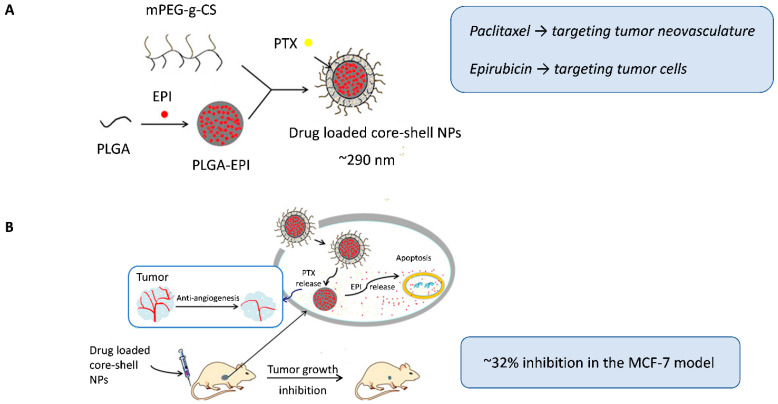
(**A**) Dual-loaded core-shell nanoparticles, with paclitaxel incorporated in the outer envelope to target tumor neovasculature and epirubicin encapsulated in the PLGA core to target tumor cells. (**B**) Schematic representation of the intracellular therapeutic mechanism of the nanoparticles. Following intravenous administration in nude mice bearing MCF-7 breast tumors, the core-shell nanoparticles achieved enhanced antitumor and antiangiogenic effects. Adapted with permission from Qin et al. [38]. Abbreviations: PLGA, Poly(lactic-co-glycolic acid); mPEG-g-CS, monomethoxy polyethylene glycol graft-chitosan; EPI, epirubicin; PTX, paclitaxel; NPs, nanoparticles.

Qin et al. [38] used another interesting strategy to encapsulate two classic anticancer drugs, but with distinct lipophilic natures, within a single nanocarrier. A double-layer core-shell system modified with mPEG-g-chitosan was designed to load paclitaxel in the outer hydrophobic envelope (targeting tumor neovasculature) and retain epirubicin in the PLGA core for delivery to tumor cells (Figure 2A). This structure enabled for controlled sequential release, with early exposure to the shell-associated antiangiogenic paclitaxel, followed by the slower release of the hydrophilic cytotoxic agent epirubicin from the core, which was consistent with the clinical sequence of the administration of antiangiogenic and cytotoxic drugs. The co-loaded core-shell nanoparticles enhanced tumor growth inhibition (~32% inhibition) and significantly reduced microvessel density in MCF-7 xenografts compared to single-loaded nanocarriers or combined free drugs, which was attributed to the prolonged circulation time and better intratumoral accumulation mediated by the nanocarrier design (Figure 2B).

### 3.2. Co-Encapsulation of Chemotherapeutics with Tumor Resistance Modulators

An emerging strategy to overcome chemotherapy resistance involves the co-delivery of a cytotoxic drug with a targeted agent that modulates tumor resistance pathways. This approach simultaneously attacks cancer cells while disabling their primary defense mechanisms, such as enhanced DNA repair, drug efflux, or an immunosuppressive tumor microenvironment. Furthermore, the well-recognized advantages of co-encapsulation are leveraged here, including the maintenance of synergistic drug ratios, improved bioavailability and tumor targeting, ultimately reducing systemic toxicity.

Zhang et al. [31] addressed platinum resistant ovarian cancer (PROC) by co-encapsulating cisplatin and wortmannin, a PI3K inhibitor that blocks DNA repair, within PLGA-PEG nanoparticles. PROC often develops resistance through reduced drug uptake and enhanced DNA repair. The formulation showed a small particle size (~100 nm) and controlled release kinetics for both drugs under physiological conditions. In vitro, the encapsulated combination presented strong synergy at different molar ratios (CI < 0.16), particularly in resistant cells (A2780cis). The developed formulation was associated with radiotherapy and tested in murine ovarian cancer xenograft models (A2780 and A2780cis), with significantly greater efficacy compared to the combination of single-drug nanoparticles or the free drug combination. The nanoparticles also enhanced radiosensitivity and increased intratumoral cisplatin-DNA adduct formation, confirming the reversal of resistance mechanisms. 

**Figure 3:**
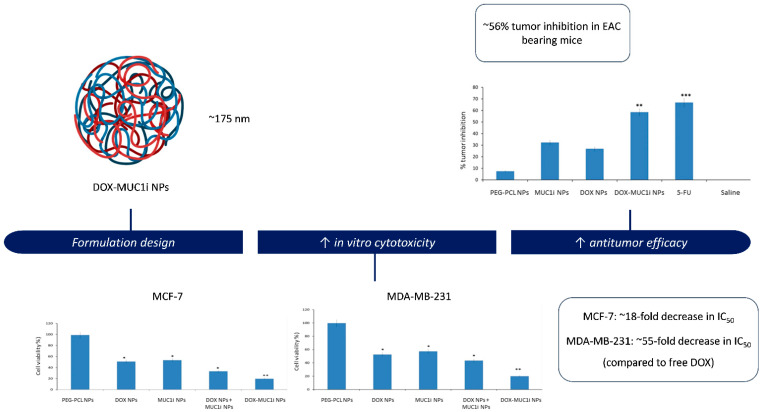
PEG-PCL nanoparticles co-delivering doxorubicin and GO-201, a MUC1 inhibitor. The formulation exhibited enhanced cytotoxicity against MCF-7 and MDA-MB-231 breast cancer cells, as well as increased antitumor efficacy in vivo in an EAC tumor model. Adapted with permission from: Behl et al. [46]. Abbreviations: DOX, doxorubicin; MUC1i, mucin 1 inhibitor; NPs, nanoparticles; IC50, half-maximal inhibitory concentration; EAC, Ehrlich ascites carcinoma; Asterisks indicate significant differences compared to PEG-PCL NPs: * *p* < 0.05; ** *p* < 0.01; *** *p* < 0.001. Beyond simple co-delivery, the combination of chemotherapeutics with resistance modulators represents a strategic shift towards functionally active nanocarrier systems. Unlike traditional systems where the polymer acts solely as a matrix, these formulations employ functional excipients (such as pluronic block copolymers) or specific inhibitors (like verapamil) to actively disrupt resistance mechanisms. By inhibiting P-gp efflux pumps while simultaneously releasing the cytotoxic payload, these systems re-sensitize MDR cells that would otherwise be impervious to treatment. In this context, the nanocarrier plays an active role in shaping therapeutic outcomes, extending beyond passive drug transport [64].

Behl et al. [46] also addressed chemoresistance and the progressive malignancy characteristic of triple-negative breast cancer (TNBC) by developing PEG–PCL nanoparticles for the simultaneous delivery of doxorubicin and GO-201, a peptide inhibitor of the oncogenic MUC1-C subunit (Figure 3). MUC1-C is overexpressed in multiple solid tumors and contributes to chemoresistance through activation of pro-survival pathways such as PI3K/Akt and NF-κB, as well as by sustaining anti-apoptotic signaling. The dual drug-loaded micelles exhibited a particle size close to 175 nm, high encapsulation efficiencies (>85%), and sustained release of both agents. In vitro assays demonstrated enhanced cytotoxicity against MCF-7 and MDA-MB-231 breast cancer cells, attributed to the synergistic activity of doxorubicin and the MUC1 inhibitor, with CI values < 1.0. Co-encapsulation also enabled coordinated intracellular delivery, resulting in increased apoptosis and suppression of MUC1-C signaling. In vivo, the dual-loaded nanoparticles produced ~56% tumor growth inhibition in the EAC model, while maintaining an acceptable safety profile as evidenced by stable body weight.

In another example, Lim et al. [37] also addressed the immunosuppressive tumor microenvironment in the TNBC through the co-administration of paclitaxel and the CSF1R inhibitor, PLX3397, within poly(2-oxazoline) (POx) polymeric micelles. TNBC tumors are frequently infiltrated with tumor-associated macrophages (TAMs) that promote immunosuppression and metastasis. The POx micelle platform enabled the formation of small particles (~57 nm) with high encapsulation efficiencies for both hydrophobic drugs (>90%). In addition, enhanced colloidal stability was attributed to interactions between the electronegative and hydrogen bond-accepting groups of paclitaxel and the amide bond motifs within POx. In vitro, strong synergy between the co-encapsulated drugs (CI < 0.3) was observed in 4T1 and T11-apobec cells across almost the entire range of the cell fraction affected. Moreover, increased antitumor activity in vivo was achieved in immunocompetent models (particularly 4T1 and T11-apobec), accompanied by an efficient suppression in the metastatic spread toward the lungs. Treatment with the formulation remodeled the tumor microenvironment by repolarizing TAMs to a pro-inflammatory phenotype (M1), depleting myeloid-derived suppressor cells, and increasing CD8+ T cell infiltration, ultimately resulting in a consistent therapeutic improvement.

### 3.3. Co-Encapsulation of Chemotherapeutics with Gene Therapy Agents

The integration of small-molecule anticancer drugs with gene therapy within a single delivery system represents an advanced evolution of combination therapy. Polymeric carriers have been used to co-encapsulate siRNA or shRNA with cytotoxic agents, enabling simultaneous modulation of gene expression and direct tumor killing. This dual mechanism allows tumor cell death pathways to be reprogrammed, enhances immunogenicity, and selectively suppresses oncogenic signaling while providing controlled pharmacokinetics and the protection of nucleic acids against degradation.

Building on this concept, the co-delivery of chemotherapeutic agents with gene-silencing therapeutics such as siRNA or shRNA has been explored as a means to sensitize tumor cells to cytotoxic stress. While chemotherapy induces DNA damage or mitotic arrest, cancer cells frequently activate compensatory survival and repair pathways that limit treatment efficacy [65]. The simultaneous delivery of nucleic acids targeting key resistance-related genes, including survivin or VEGF, can attenuate these adaptive responses during drug exposure. As a result, such combination systems may enhance therapeutic efficacy at reduced drug doses and contribute to delaying or mitigating the emergence of treatment resistance.

In this context, Jin et al. [23] developed pH-responsive polymeric nanoparticles with PEG-detachable properties for the co-delivery of paclitaxel and siRNA against the survivin gene for lung cancer therapy. The system was constructed from a cationic PEI-PLA copolymer that self-assembled into nanoparticles, in which the hydrophobic PLA core encapsulated paclitaxel while the PEI shell complexed survivin siRNA through electrostatic interactions. An anionic PEG-PAsp copolymer was further coated on the nanocarrier surface to provide stealth characteristics during circulation. After nanoparticle uptake by tumor cells, the PEG-PAsp segments became electrically neutral due to the acidic endosomal pH and consequently detached, exposing the cationic PEI, which facilitated endosomal escape via the proton sponge effect and enabled cytosolic release of both paclitaxel and siRNA. The optimized formulation presented a particle size of ~82 nm, high encapsulation efficiency of paclitaxel (~93%), and great colloidal stability in PBS and PBS supplemented with 10% FBS. In vitro and in vivo studies in A549 lung cancer models showed that this co-delivery system effectively knocked down survivin expression, induced significant apoptosis and cell cycle arrest, and resulted in superior tumor growth inhibition, ultimately extending survival compared to the control treatments.

**Figure 4:**
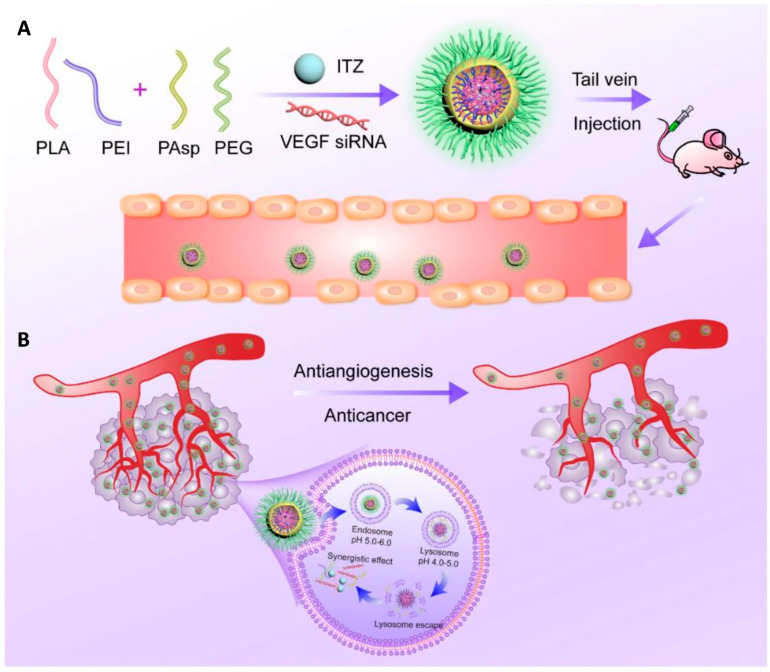
(**A**) Nanoparticle delivery system co-loading itraconazole and VEGF siRNA. (**B**) Schematic representation of the intracellular therapeutic mechanism of the co-loaded nanoparticles, which can synergistically inhibit tumor angiogenesis and thereby exert an antitumor effect against breast cancer. Reprinted from: Jin et al. [23]. Abbreviations: PLA, poly(lactic acid); PEI, polyethyleneimine; PAsp, poly(L-aspartic acid sodium salt); PEG, poly(ethylene glycol); ITZ, itraconazole; VEGF siRNA, small interfering RNA targeting vascular endothelial growth factor.

In a subsequent study, the same research group applied this platform to co-deliver repurposed itraconazole and VEGF siRNA for breast cancer therapy. Itraconazole is a conventional antifungal drug with a well-established safety profile that has recently been repurposed as a multi-target antiangiogenic agent for cancer treatment, while VEGF is one of the key angiogenic mediators in tumor progression. The nanoparticles, again based on the PEI-PLA/PEG-PAsp system, successfully co-loaded the highly hydrophobic itraconazole into the PLA core, whereas the hydrophilic siRNA was electrostatically complexed with the cationic PEI shell (Figure 4A). The formulation exhibited a suitable particle size (~118 nm), good colloidal stability, and pH-responsive drug release. In vitro and in vivo studies in 4T1 breast cancer models demonstrated that the co-delivery system effectively silenced VEGF expression, suppressed angiogenesis and tumor cell migration, and induced significant apoptosis. Moreover, the combination therapy showed superior antitumor efficacy due to the combined antiangiogenic and antitumor effects (Figure 4B), while maintaining a low toxicity profile [35]. 

Beyond co-delivering chemotherapeutics and gene modulators, an additional design that further enhances therapeutic precision involves the incorporation of active targeting ligands into the nanocarrier surface. While the enhanced permeability and retention (EPR) effect relies on passive and highly heterogeneous tumor accumulation, ligand-mediated surface functionalization, using moieties such as folate or mannose, enables receptor-mediated endocytosis and more selective cellular uptake. This strategy is particularly relevant for metastatic or circulating tumor cells, which often lack a well-defined solid tumor microenvironment [9,18,66].

In line with this strategy, Chen et al. [47] developed folate-targeted pullulan-based nanomicelles capable of co-delivering doxorubicin and shRNA against Beclin-1 to simultaneously induce tumor cell apoptosis and suppress autophagy-mediated chemoresistance. In this nanosystem, hydrophobic desoxycholic acid domains enabled efficient doxorubicin encapsulation within the micellar core, while low molecular weight PEI conjugation facilitated electrostatic complexation of shBeclin-1. Folate functionalization further promoted selective internalization via folate receptor-mediated endocytosis. The formulation displayed a small particle size (~162 nm) and pH-responsive doxorubicin release, together with higher cytotoxicity and enhanced apoptotic induction in folate-positive HeLa cells. In vivo, a remarkable antitumor efficacy was observed in HeLa tumor-bearing mice, with almost no tumor growth throughout the treatment period, confirming that autophagy blockade promoted by Beclin-1 silencing can significantly potentiate doxorubicin efficacy while maintaining low systemic toxicity.]

## 4. Targeted and Stimuli-Responsive Nanoparticles for Tumor Drug Co-Delivery

The high incidence of adverse effects associated with the nonspecific action of conventional chemotherapeutic drugs has driven the search for alternatives capable of directing drug activity toward target cells, thereby improving both efficacy and safety. In this context, nanotechnology offers promising approaches to promote the targeted delivery of therapeutic agents to tumor tissues, enabling preferential drug accumulation at the site of action. Such targeting can be achieved through passive or active mechanisms.

**Figure 5:**
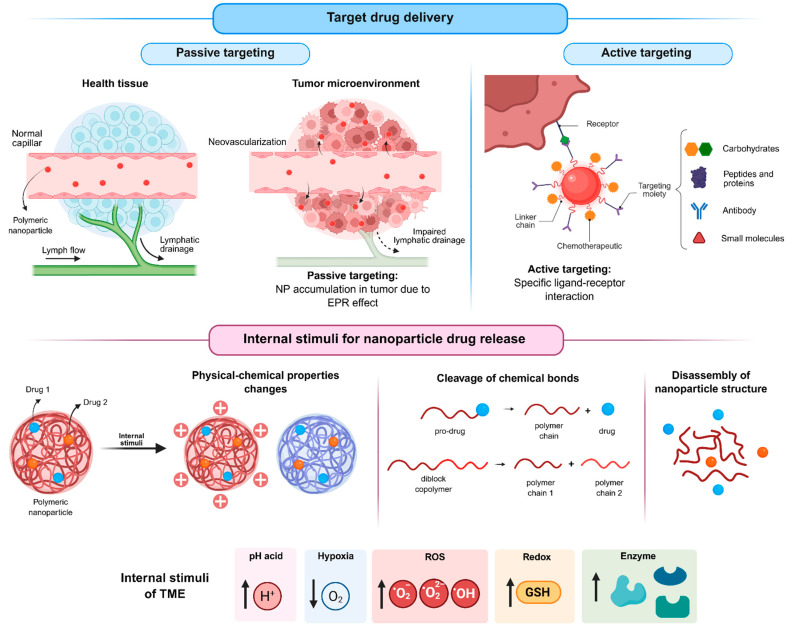
Strategies of targeting for nanoparticles and drug release stimulated by the tumor microenvironment (created in BioRender. De Oliveira, M.A. (2026) https://BioRender.com/ugp6mnk, accessed on 19 January 2026).

Passive targeting (Figure 5) is based on the distinctive physiological features of tumor tissues. The high metabolic rate of tumors induces angiogenesis, however, the newly formed blood vessels are typically abnormal and poorly organized due to a lack of proper maturation. As a result, these vessels present fenestrations that allow nanoparticles to extravasate into the tumor interstitium, favoring their local accumulation. Moreover, the deficient lymphatic drainage characteristic of tumor tissues hinders nanoparticle clearance, enabling their prolonged retention at the tumor site. The combination of increased vascular permeability and impaired lymphatic drainage is known as the EPR effect [67,68]. 

In contrast, active targeting (Figure 5) relies on the surface functionalization of nanoparticles with biological ligands, such as small molecules [47,49,55], peptides and proteins [60], carbohydrates [39,50], or antibodies, which can specifically recognize receptors overexpressed on tumor cells. The resulting ligand–receptor interactions enhance selectivity and cellular uptake, thereby improving therapeutic efficacy while minimizing off-target effects on healthy tissues.

Beyond targeting strategies, controlled drug release triggered by tumor-specific stimuli (Figure 5) represents another important approach to further enhance the selectivity of antitumor therapies. Several characteristics of the tumor microenvironment, including acidic pH, hypoxia, abnormal enzyme expression, elevated reactive oxygen species (ROS) levels, and altered redox potential, can be exploited for this purpose.

Among the internal stimuli, the acidic pH of the tumor microenvironment is one of the most extensively investigated triggers for drug release from stimulus-responsive nanoparticles (srNPs). Tumor cells predominantly generate energy through glycolysis rather than oxidative phosphorylation, converting glucose into lactic acid even under aerobic conditions, a phenomenon known as the Warburg effect [69,70]. Consequently, tumors exhibit a lower extracellular pH (approximately 6.5) compared with normal tissues (approximately 7.4), with even more acidic conditions found in intracellular organelles such as endosomes and lysosomes [71].

pH-responsive nanoparticles are designed using components that undergo structural and/or chemical changes under acidic conditions. These changes destabilize the nanoparticle structure, leading to drug release. Although the acidic tumor environment is advantageous for triggering drug delivery from srNPs, it is also associated with tumor progression and drug resistance, particularly to weakly basic drugs, such as doxorubicin [72,73].

As an example, Dong et al. [42] developed pH-sensitive micelles for the co-delivery of docetaxel and silibinin. For this purpose, the authors synthesized a novel block copolymer, polyethylene glycol-block-poly[(1,4-butanediol)-diacrylate-β-N,N-diisopropylethylenediamine] (PEG-b-BDP). Under acidic conditions (pH < 6.3), the BDP block undergoes a transition from a hydrophobic to a hydrophilic state as a result of the protonation of the β-N,N-diisopropylethylenediamine moieties. This physicochemical change destabilizes the micellar structure, leading to micelle dissociation and subsequent release of the encapsulated drugs into the endosomal/lysosomal compartments of tumor cells. 

Another strategy reported for the development of pH-responsive nanoparticles in the reviewed studies involves the use of polymers containing chemical bonds that are susceptible to cleavage under mildly acidic conditions. This approach was adopted by Cheng et al. [34], who employed poly(ortho ester urethane) copolymers, and by Zhang et al. [30], who utilized amphiphilic poly(β-amino ester) copolymers for nanoparticle development. Similarly, Zhang et al. [54] conjugated carboplatin to the polymer PEG-PLGA and subsequently developed self-assembled nanoparticles for the co-administration of paclitaxel and carboplatin. The resulting nanoparticles exhibited pH-dependent drug release behavior. Specifically, the amide bond formed between the carboplatin molecule and the PEG-PLGA chain is susceptible to acid-catalyzed hydrolysis, which promotes drug release in acidic environments, such as those found within tumor cells, particularly in endosomes and lysosomes, as well as in the tumor microenvironment. Furthermore, the PLGA segment itself undergoes hydrolytic degradation under low pH conditions, further facilitating the degradation of the nanoparticles and increasing the release of their encapsulated contents. 

Another important characteristic of solid tumors is hypoxia, which arises from abnormal vascularization and insufficient oxygen supply. Oxygen levels in tumor tissues are significantly lower (around 2%) than in normal tissues (around 9%) and may approach zero in poorly perfused regions, such as in deeper regions of the tumor. Hypoxia is considered an important prognostic marker and is strongly associated with tumor progression, metastasis, angiogenesis, and drug resistance. Tumor cells under hypoxic conditions often exhibit reduced proliferation rates, making them less sensitive to chemotherapeutic agents that primarily target rapidly dividing cells. Furthermore, hypoxia promotes the activation of hypoxia-inducible factors, which regulate genes involved in tumor growth, metastasis, and angiogenesis [74].

Despite its role in cancer progression, hypoxia can be exploited as a stimulus for the development of responsive drug delivery systems. Hypoxia-responsive nanoparticles commonly incorporate polymers with reducible chemical groups, such as azo bonds, nitrobenzyl alcohols, and nitroimidazoles. Under low-oxygen conditions, these groups undergo bioreduction, leading to changes in the physicochemical properties of the carrier, nanoparticle destabilization, and subsequent drug release.

Hypoxia in the tumor microenvironment also significantly affects ROS levels. Tumor cells typically produce higher amounts of ROS than normal cells due to oncogene activation, chronic inflammation, and mitochondrial dysfunction. While ROS play essential roles in cellular signaling at physiological levels, their elevated concentrations in tumors provide an effective endogenous trigger for selective drug release. ROS-responsive nanoparticles contain chemical moieties that are sensitive to oxidative conditions, such as thioethers, selenides, arylboronic acid esters, thioacetals, aminoacrylates, peroxalate esters, etc. In these systems, drug release can occur through ROS-induced nanoparticle degradation, cleavage of the polymer backbone, or rupture of polymer-drug linkages [75,76,77].

Wu et al. [52] synthesized the amphiphilic polymer methoxy poly(ethylene glycol)-block-poly[(N-2-hydroxyethyl)-aspartamide] (mPEG-b-PHEA) and functionalized its hydrophobic segment with phenylboronic acid (PBA) moieties to obtain self-assembling polymeric micelles capable of co-encapsulating the antitumor drugs doxorubicin and irinotecan. In this system, PBA acted as an electron acceptor, forming donor–acceptor coordination bonds with doxorubicin and irinotecan, which contributed to drug loading and micellar stability. Due to the reversible nature of these coordination interactions and the high sensitivity of PBA to ROS, particularly hydrogen peroxide, the micelles exhibited ROS-responsive behavior. This property enabled efficient and selective drug release in tumor cells, where ROS levels are typically elevated, thereby enhancing the site-specific delivery of both drugs.

Enzyme-responsive delivery systems take advantage of the abnormal expression of specific enzymes in tumor tissues. Compared with normal cells, tumor cells frequently overexpress enzymes such as matrix metalloproteinases (MMPs), hyaluronidases, esterases, and β-glucosidases, resulting in elevated enzyme concentrations within the tumor microenvironment. Enzyme-responsive nanoparticles incorporate substrates that can be selectively cleaved by these enzymes. Enzymatic activity destabilizes the nanoparticle structure, for instance, through polymer chain cleavage or polymer–drug linkage, thereby enabling site-specific drug release within the tumor microenvironment.

Another distinguishing feature of tumor cells is their altered redox potential, characterized by elevated intracellular levels of glutathione (GSH). GSH is a thiol-containing tripeptide composed of glutamate, glycine, and cysteine and represents one of the most abundant intracellular antioxidants. It plays a central role in maintaining redox homeostasis, detoxification processes, immune function, and protection against oxidative stress. In addition, GSH is responsible for the cleavage of disulfide bonds through redox reactions. The significant differences in GSH concentrations between the bloodstream, normal cells, and tumor cells have been widely exploited in the design of redox-responsive drug delivery systems. These systems typically incorporate disulfide or diselenium bonds, which remain stable under extracellular conditions but are rapidly cleaved in the presence of high intracellular GSH concentrations, leading to nanoparticle destabilization and selective drug release within tumor cells [78].

With the aim of developing increasingly sophisticated and intelligent nanoparticles, several studies have integrated multiple strategies to achieve more efficient and selective drug delivery to the tumor site. In addition to the co-delivery of drugs with distinct mechanisms of action, approaches such as active targeting and stimulus-responsive release have been combined to further enhance therapeutic efficacy and specificity toward tumor tissues.

Wang et al. [60] developed a multifunctional nanocarrier system for the targeted co-delivery of adavosertib and olaparib in ovarian cancer therapy. The nanoparticles, prepared from mesoporous polydopamine (PDA), were surface-decorated with the TMTP1 peptide, whose molecular target is the aminopeptidase P (XPNPEP2), a receptor that is highly expressed on the cell membrane of ovarian cancer cells, thereby enabling active targeting. In addition to its targeting capability, polydopamine exhibits physicochemical properties that support stimulus-responsive drug release in the tumor microenvironment. The π–π interactions established between PDA and the drugs can be weakened by protonation of the amino groups of PDA under acidic conditions, as well as by the action of GSH, resulting in accelerated drug release at the tumor site. Furthermore, the abundant phenolic hydroxyl groups present in PDA can be oxidized by hydrogen peroxide, which reduces the hydrogen-bonding interactions between the drugs and the polymeric matrix. Therefore, besides the active tumor targeting mediated by the TMTP1 peptide, the developed nanoparticles display multi-stimuli-responsive drug release behavior, being sensitive to key characteristics of the tumor microenvironment, including acidic pH, redox potential, and elevated levels of ROS.

Han et al. [50] combined mannose-mediated active targeting with pH-responsive drug release in the development of PLGA-based nanoparticles for the co-encapsulation of plumbagin, dihydrotanshinone I, and ammonium bicarbonate (NH_4_HCO_3_). Coating the nanoparticle with a mannose-enriched erythrocyte membrane resulted in a biomimetic system capable of selectively targeting hepatocellular carcinoma cells, which overexpress mannose receptors. Simultaneously, the incorporation of ammonium bicarbonate conferred pH-sensitive release properties to the nanocarrier. Under acidic conditions, ammonium bicarbonate undergoes decomposition, generating NH_3_ and CO_2_, which induces nanoparticle disruption, thereby promoting the rapid and localized release of the encapsulated antitumor agents. 

Chen et al. [47] modified pullulan, a natural homopolysaccharide, with lipophilic deoxycholic acid, PEI, and folic acid (FA) to obtain a multifunctional copolymer. This folate-functionalized amphiphilic bifunctional pullulan-based copolymer (FPDP) was employed in the fabrication of polymeric micelles for the co-encapsulation of doxorubicin and shBeclin1. The incorporation of folic acid on the nanoparticle surface enabled active targeting toward tumor cells that overexpress folate receptors. The presence of lipophilic deoxycholic acid promoted the efficient encapsulation of doxorubicin through hydrophobic interactions, while the cationic nature of PEI facilitated the complexation of the negatively charged shBeclin1 via electrostatic interactions. Moreover, under acidic conditions, the amino groups of PEI undergo protonation, which alters the physicochemical properties of the FPDP polymer and destabilizes the micellar structure. This pH-responsive behavior enhances drug release, thereby facilitating the selective delivery of doxorubicin in the tumor microenvironment.

Wang et al. [55] also employed folic acid as a targeting ligand in the design of nanoparticles for cancer therapy, combining active targeting with pH- and redox-responsive drug release of doxorubicin and paclitaxel. The nanoparticles were constructed using a triblock copolymer composed of PLA, PEI, and PEG. Disulfide bonds incorporated between the polymer blocks endowed the system with redox sensitivity, as these linkages can be cleaved in response to the elevated intracellular levels of GSH typically found in tumor cells. This reduction-triggered cleavage results in polymer degradation and subsequent release of the encapsulated drugs. In addition, a doxorubicin prodrug was synthesized by conjugating the drug to 2,3-dimethylmaleic anhydride through an acid-labile bond. Under acidic conditions, characteristic of the tumor microenvironment, this linkage is cleaved, enabling the selective release of doxorubicin. Acidic pH also induced a change in the surface charge of the nanoparticle, rendering them positively charged, which may enhance cellular uptake through electrostatic interactions with the negatively charged cell membrane. Furthermore, the presence of PEI in the polymeric structure facilitated endosomal/lysosomal escape via the proton-sponge effect, thereby improving the intracellular availability of the delivered drugs. Overall, the nanoparticles developed by the authors integrated multiple tumor-specific physiological triggers, including receptor-mediated targeting, acidic pH, and redox potential, to achieve a highly selective and efficient delivery system for combination cancer therapy.

## 5. Challenges and Limitations of Polymeric Co-Delivery Systems in Cancer Therapy

Despite the great potential of polymeric co-administration systems, their clinical translation remains hampered by multifactorial challenges. These limitations mainly involve biological, manufacturing, and regulatory aspects, and must be systematically addressed for these technologies to succeed in the clinic. Furthermore, although co-encapsulation is frequently presented as a strategy to achieve synergistic anticancer effects, the present analysis indicates that synergy is often assumed rather than rigorously demonstrated, as nearly half of the reviewed studies did not calculate a CI or equivalent quantitative metric.

One of the main biological obstacles to the clinical translation of polymeric nanocarriers is the inefficient and heterogeneous delivery of nanoparticles to the target tumor site. The EPR effect, while historically regarded as a cornerstone of nanomedicine, is highly variable among human patients and often far less pronounced than in preclinical animal models [79,80]. In practice, many nanoparticle delivery systems accumulate only a tiny fraction of the injected dose (<10%) in human tumors via passive targeting [81]. This variability exists because EPR-mediated extravasation is heavily influenced by factors specific to each tumor and patient, such as vascular architecture, interstitial fluid pressure, stromal density, and immune cell infiltration within the tumor microenvironment [79,82]. Furthermore, physiological barriers like rapid clearance by the mononuclear phagocyte system further cut down nanoparticle circulation time, hinder deep tumor penetration, and prevent an even distribution within the tumoral tissue [83].

**Figure 6:**
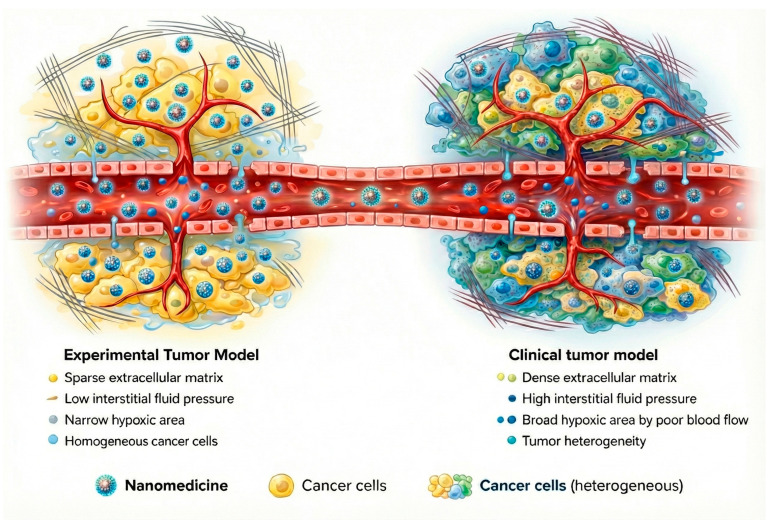
Schematic comparison between experimental tumor models and human tumors highlighting key factors influencing nanoparticle delivery.

Growing clinical evidence indicates that the contribution of the EPR effect to effective tumor targeting has frequently been overestimated. Preclinical models tend to overpredict nanoparticle accumulation because animal tumors differ fundamentally from human cancers in their size, vascularization, and growth dynamics (Figure 6) [79,82,84]. As a result, reliance on passive targeting alone has shown limited predictive value for clinical outcomes. While adding active targeting ligands, such as antibodies or PSMA-targeting moieties, can improve tumor selectivity, these approaches introduce their own complexities, including heightened immunogenicity and manufacturing challenges [66]. Given these limitations, the focus is now shifting to more comprehensive design strategies that integrate combined pharmacological and physical treatments to prepare tumors for better delivery and efficacy through the use of multi-stage and/or stimulus-responsive delivery nanomaterials and the combination of nanotherapies with immunotherapy [82].

From a manufacturing perspective, achieving high encapsulation efficiency and drug loading for multiple agents with divergent physicochemical properties remains particularly challenging, although some systems discussed herein have shown consistent results at a laboratory scale. Moreover, ensuring that the synergistic drug ratios are maintained during manufacturing, storage, and after administration is also a major challenge. Premature drug leakage during storage or circulation may lead to systemic toxicity and prevent the maintenance of the intended synergism [81].

The safety profile of the materials used in polymeric carriers also requires attention. Although many polymers have been designed for co-delivery, only a small number have reached regulatory approval by the FDA. Biodegradable polymers, such as PLGA, are widely preferred; however, the potential for the long-term accumulation of degradation products or residual synthetic components remains uncertain [62]. Cationic polymers commonly used for gene co-delivery can induce cytotoxicity and immune responses, limiting their applicability. Furthermore, some manufacturing techniques, although effective for producing co-delivery systems, still rely on toxic organic solvents, which may restrict their application [81].

Overall, while the rationale for co-delivery using polymeric systems is solid, the transition from promising preclinical data to clinical success still requires concerted efforts to overcome all of these challenges.

### The Brazilian Landscape: Bridging the Gap Between Innovation and Translation

As the leading producer of scientific knowledge in Latin America, Brazil offers a good perspective on the global challenges of nanomedicine translation. The country has established a robust academic foundation for nanocarrier development, supported largely by the National System of Laboratories in Nanotechnologies (SisNANO). Created to foster collaboration between public universities and the private sector, SisNANO provides high-level infrastructure for the characterization and preclinical validation of complex polymeric systems, mitigating the equipment deficit often faced in emerging economies.

However, a distinct “valley of death” remains between academic innovation and market access. While Brazilian universities demonstrate high productivity in publishing data on co-loaded polymeric nanoparticles, the conversion of these findings into patents and commercial products is disproportionately low compared to North American and European counterparts. This discrepancy is often attributed to a historical disconnection between academia and the pharmaceutical industry, as well as the high cost of importing specialized GMP-grade raw materials required for clinical manufacturing.

From a regulatory standpoint, the Brazilian Health Regulatory Agency (ANVISA) has made significant strides in aligning its standards with international bodies like the IMDRF. The recent Resolution RDC 751/2022 formally defines nanomaterials for medical devices, harmonizing Brazil’s framework with global best practices. Nevertheless, the specific regulatory pathway for complex nanomedicines (non-biological complex drugs) is still evolving. The lack of specific “nano-regulation” often forces a case-by-case analysis that can slow the approval process for innovative co-delivery formulations. For the field to advance locally, there is an urgent need to strengthen the triple helix model, integrating government funding, academic research, and industrial capability to ensure that the sophisticated co-delivery systems designed in Brazilian laboratories can successfully navigate the regulatory pathway and reach clinical application.

## 6. Commercial Status and Clinical Translation

Although the vast majority of polymeric nanocarriers described in this review are in preclinical development, it is important to acknowledge that single-agent polymeric nanoparticles have successfully reached the global market. The most prominent example is Genexol-PM^®^ (Cynviloq), a polymeric micelle formulation of paclitaxel (poly(ethylene glycol)-block-poly(D,L-lactide)) approved in South Korea and under evaluation in Europe for the treatment of breast and lung cancers [85]. Other commercially available formulations include Nanoxel^®^, a polymeric paclitaxel nanoparticle approved in India [86], and Apealea^®^ (paclitaxel micellar), which received European marketing authorization for ovarian cancer [87]. These successes validate the safety and manufacturing feasibility of polymeric nanocarriers for human use.

However, a critical gap remains in the translation of co-loaded systems. To date, there are no regulatory-approved polymeric nanoparticles co-loading two or more chemotherapeutic agents on the market [88]. While “cocktail” chemotherapy (administering two distinct drugs) is standard clinical practice, the fixed-dose co-encapsulation of synergistic pairs in a single polymeric vehicle, as extensively reviewed here, remains stuck in preclinical or early clinical phases. This discrepancy between the clinical success of single-drug polymeric carriers and the absence of co-loaded counterparts highlights the complexity of validating combination nanomedicines, where challenges in ratio control, batch-to-batch reproducibility, and complex regulatory requirements for combination products must still be overcome.

## 7. Future Perspectives for Polymeric Co-Delivery Systems

The future of polymeric co-administration systems remains highly promising, despite current limitations. Intelligent, stimulus-responsive polymeric architectures capable of reacting to biochemical signals in the tumor microenvironment (such as pH, redox states, and enzymatic activity) may allow for synchronized and site-specific release of co-encapsulated agents, enabling better maintenance of synergistic drug ratios in vivo [23,35]. In parallel, modular polymeric designs adapted to accommodate molecules with divergent physicochemical properties may reduce competitive encapsulation effects and improve formulation stability [62].

In recent years, advances in nanomedicine have increasingly emphasized that tumor accumulation and drug co-encapsulation alone are insufficient to guarantee therapeutic success. The rational modulation of nanocarrier physicochemical parameters, including particle size, surface chemistry, and stealth properties, can be strategically leveraged to control circulation time, biodistribution, and clearance pathways, thereby improving tumor exposure while minimizing off-target accumulation and systemic toxicity [89]. Notably, in co-loaded systems, coordinated pharmacokinetics are essential to preserve the intended drug ratios at the target site, which represents a critical prerequisite for sustained synergistic activity in vivo.

Beyond systemic delivery, a growing body of evidence shows that intracellular barriers are decisive in determining the success of nanocarrier-based therapies. Factors such as the cellular uptake pathway, endosomal trafficking, escape efficiency, and final subcellular localization ultimately control whether the delivered agents ever reach their molecular targets [90,91]. In this context, polymeric nanocarriers engineered to promote controlled intracellular release or organelle-specific targeting may enhance drug synergy, overcome multidrug resistance, and improve treatment durability. These emerging concepts suggest that future development of polymeric co-delivery systems should integrate pharmacokinetic control with precise regulation of intracellular fate, thereby helping to bridge the gap between promising preclinical outcomes and successful clinical translation [90].

A particularly relevant future direction involves the co-delivery of chemotherapeutic agents with tumor resistance modulators, such as drug efflux pump inhibitors, metabolic pathway regulators, or signaling modulators involved in resistance adaptation. Polymeric carriers also represent an attractive platform for gene co-delivery strategies, enabling the combined administration of nucleic acid therapies (siRNA, shRNA) with conventional small molecules. These combinatorial approaches may allow the simultaneous suppression of resistance pathways at the transcriptomic or proteomic level while administering cytotoxic agents or targeted molecules to the same cell population [35,47]. The integration of diagnostic and therapeutic functions, known as theranostics, is also a promising, yet largely unexplored, prospect. Polymeric nanoparticles can be simultaneously loaded with therapeutic agents and contrast agents for imaging, allowing for real-time monitoring of drug release, accumulation, and treatment response [92].

Future progress will also depend on advances in scalable manufacturing methods, including continuous-flow microfluidic manufacturing and solvent-free approaches that minimize toxic processing steps. Research should additionally focus on ensuring long-term stability and conducting comprehensive safety and toxicology studies. The use of biodegradable and FDA-approved polymers (e.g., PLGA, PEG, PLA) will be crucial to facilitate regulatory approval [81]. Furthermore, the integration of computational modeling, AI-guided formulation design, and predictive models for pharmacokinetics and biodistribution may accelerate the rational selection of optimal drug ratios and guide more efficient development processes [93].

Innovations in responsive materials, strategies to modify and overcome resistance mechanisms, gene co-delivery approaches, scalable manufacturing, and data-driven formulation design are expected to shape the next generation of polymeric co-delivery systems, potentially enabling these platforms to achieve consistent clinical benefits. 

Overall, this review reframes polymeric co-delivery systems not merely as advanced drug carriers, but as therapeutic tools whose value must be judged by measurable biological and preclinical outcomes. By highlighting inconsistencies in synergy assessment, experimental design, and safety evaluation, and by proposing a mechanism-driven framework for interpreting co-delivery strategies, this work aims to support more reproducible, translationally relevant research. Such an approach is essential to bridge the gap between promising preclinical results and successful clinical implementation of polymeric nanocarriers for combination chemotherapy.

## 8. Conclusions

This review provides added value to the current literature by moving beyond descriptive summaries of polymeric nanocarriers and offering a critical, outcome-oriented perspective on combination chemotherapy. Several reviews have addressed polymeric nanocarriers for combination cancer therapy, primarily emphasizing formulation strategies, polymer chemistry, or carrier classification, with limited critical appraisal of therapeutic outcomes [62,94,95]. In particular, a prevailing assumption in the field is that co-encapsulation of multiple drugs within a single polymeric nanocarrier intrinsically leads to synergistic anticancer effects. However, recent analyses have highlighted that synergy is frequently presumed rather than rigorously demonstrated, as quantitative pharmacological metrics such as the CI are often omitted [96,97]. Consistent with these observations, our analysis reveals that nearly half of the preclinical studies published in the last decade do not formally assess synergy, despite reporting improved efficacy.

Moreover, existing reviews rarely address the substantial heterogeneity in tumor models, dosing regimens, efficacy endpoints, and toxicity assessment, which limits cross-study comparison and weakens translational interpretation [98,99]. To address these gaps, the present review adopts an outcome-oriented perspective and introduces a functional, mechanism-driven framework. By linking nanocarrier design features to their underlying biological rationale, this framework provides a structured basis for interpreting preclinical efficacy and guiding the rational development of clinically relevant polymeric co-delivery platforms.

## 9. Material and Methods

Schematic illustrations presented in Figure 1 and Figure 6 were generated using a generative artificial intelligence model (Gemini 3.0 Pro, Google DeepMind) for graphical representation purposes only. Textual prompts were made using the figure’s caption plus a description of polymeric nanocarriers, tumor microenvironment features, therapeutic mechanisms, and experimental versus clinical tumor models by the authors.

The generated images were reviewed for scientific accuracy and subsequently refined to improve clarity, labeling, and resolution. The figures do not contain experimental data, patient information, or quantitative analyses and were created solely as conceptual visual aids. Full responsibility for the scientific content and interpretation of the figures lies with the authors.

## Figures and Tables

**Table 1:** Highlights from in vitro and in vivo studies on co-encapsulated drugs targeting cancer in the last decade.

Cancer Targeted	Active/ Drug 1	Active/ Drug 2	Nano-Carrier Type	Method of Targeting/Release	Combination Index	In Vitro Effect of NP w/Drug Combination	Dose/Therapeutic Scheme	Adm Route	In Vivo Effect of NP w/Drug Combination	Toxic Effect	Ref.
Breast	Pyrrolidine dithio carbamate	Doxorubicin	Nano sphere	Passive targeting pH-sensitive release	ND	71.3% of growth inhibition of MCF7	6 mg/kg eq, not clear frequency of administration	IV	82.9% of tumor growth inhibition	None	[34]
	VEGF siRNA	Itraconazole	Nano sphere	Passive targeting	ND	NP combination exhibited the highest cell inhibition rate in cell proliferation assay	Itraconazole 10 mg/kg, VEGF siRNA 3 mg/kg, every 3 d/4x	IV	Best tumor inhibition, plus a decrease in angiogenesis	None reported	[35]
	Docetaxel	Thymoquinone	Nano capsule	Passive targeting	<1 at 5 µM of docetaxel	NPs produced a pronounced effect on the inhibition of migratory potential of MDA-MB-231 cells, plus 20.1 ± 3.70% of wound healing.	2 mg/kg Docetaxel and 4 mg/kg thymoquine, every 3 d/4x	IV	2.85-fold decrease in tumor volume	Slight increase in ALT and AST	[36]
	Paclitaxel	CSF1R inhibitor	Micelle	Passive targeting	<<1 at paclitaxel: CSF1R 4:1–1:4	NPs were substantially more active than either of the single drugs	75 mg/kg paclitaxel and 75 mg/kg CSF1R, every 4 d/4x.	IV	Increased apoptosis in the tumor tissue, significantly decreased the levels of metastases in lung tissues	Not reported	[37]
	Paclitaxel	Epirubicin	Core-Shell NP	Passive targeting	ND	Significant synergistic growth-inhibitory effects on the MCF-7 and HUVECs cell lines	Epirubicin 2.5 mg/kg and paclitaxel of 1.5 mg/kg every 5 d	IV	Tumor was inhibited 32%. Stronger inhibitory effect on microvessel growth.	Not reported	[38]
	Histamine	Paclitaxel	Micelle	Active targeting (Glucose)	ND	Reduced cell migration after 24 h of treatment	Histamin 12 mg/kg and paclitaxel 10 mg/kg, 3x a week/2 weeks	IV	Delineated a trend to reduce the tumor	None	[39]
	Salinomycin	Doxorubicin	Nano sphere	Passive targeting	<1 Salinomycin:Doxorubicin 1:3	Higher cancer inhibitory potential of the combined treatments of free combination or co-loaded NPs	Salinomycin 1 mg/kg and doxorubicin 3 mg/kg, twice a week/3 weeks	IV	97% tumor reduction. Inhibited the tumor reoccurrence and superior anticancer activity over the single drug loaded NPs formulations	No liver toxicity	[40]
	Salinomycin	Doxorubicin	Micelle	Passive targeting	ND	Escape from the drug efflux of A/MCF-7 cells and penetration into 3D-cultured mimic tumor spheres more effectively	Doxorubicin:salinomycin 5 mg/kg, every two d/16 d	IV	Presented a stronger solid tumor growth inhibition effect than all of the other treatment groups, especially showing an ongoing inhibition effect after drug withdrawal. Also inhibited metastasis.	None	[41]
	Docetaxel	Silibinin	Micelle	Passive targeting pH-sensitive release	ND	Depress the invasive and migratory capabilities of 4T1 cells, achieving inhibition rates of 86.9% and 88.4%	Docetaxel 4 mg/kg and silibinin 8 mg/kg, 2x w/2 w	IV	Tumor inhibition of 71.9%	Not reported	[42]
	Paclitaxel	Doxorubicin	Micelle	Passive targeting	ND	Induce MDR cancer cells arrested mainly in S and G2/M phase, indicating the potential of this nanocarrier to overcome MDR. Plus, reduction in volume of tumor spheroids at different micelle concentrations	Doxorubicin: paclitaxel 2:3 at 5 mg dose total, 3x (d 0, 4 and 8)	IV	Inhibitory tumor rate of 63.84%	No weight change	[43]
	Curcumin	Doxorubicin	Micelle	Passive targeting	ND	Higher intracellular concentration of doxorubicin. Significantly enhance the cytotoxicity, cellular uptake and cell apoptosis	Doxorubicin 10 mg/kg every other 2 d	IV	Loss of tumor weight (around 50%)	No weight change	[44]
	Hydroxycamptothecin	Ursolic Acid	Micelle	Active targeting (galactose residues in the pectin chain)	<1	Maintained a high concentration in the cytoplasm region	10 mg/kg of each, 5x (d 0, 2, 4, 6, and 8)	IV	Tumor volumes were extremely smaller than those treated with controls	No weight change	[45]
	Mucin1 glycoprotein	Doxorubicin	Nano Sphere	Passive targeting	<1	Synergistic effect on breast cancer cell death	10 mg/kg of each/9 days	IP	Tumor inhibition of 55.5%	None	[46]
Cervical	Doxorubicin	shRNA of Beclin1	Micelle	Active targeting (Folate)	ND	Effectively target FR-positive cancer cells via the FR-mediated endocytosis process. Potential to be an idealistic shRNA delivery carrier for gene silencing in the treatment of cancer.	Doxorubicin 4 mg/kg and shRNA beclin1 4.98 mg/kg, each 2 d.	IV	Highest efficiency in inhibiting tumor growth than controls	None	[47]
Colon	Paclitaxel Prodrug	Combretastatin A4	Micelle	Passive targeting	ND	Increased cytotoxicity against both CT26 and 4T1 cancer cells	Combretastatin A 16 mg/kg, paclitaxel prodrug 10 mg/kg, 4x (d 0, 3, 6 and 9)	IV	Tumor inhibition with 87.2%. NPs accumulated at the tumor site. which was significantly higher than other groups	None	[48]
Liver	Doxorubicin	Plumbagin	Nano sphere	Active targeting (Aminoethyl anisamide)	ND	significantly (*p* < 0.01) attenuated the Hepa1-6-R cell migration (20% of relative migration area)	Doxorubicin 12 mg/kg and plumbagin 2 mg/kg. Number of d not clear	IV	Improved tumor suppression and reduced tumor weight	No weight loss	[49]
	Curcumin	Doxorubicin	Nano sphere	Passive targeting pH-sensitive release	<1 at doxorubicin: Curcumin 1:10 and 1:20, molar ratio	Increased doxorubicin intake. Potent proliferation suppression activity	Doxorubicin 1 mg/kg and curcumin of 10 mg/kg, every 2 d/2 weeks	IV	Tumor inhibition of 73.37%	Barely systemic toxicity	[30]
	Plumbagin	Dihydrotanshinone I	Nano capsule	Active targeting (mannose-inserted erythrocyte membrane)	<1 at different proportions	Inhibited the proliferation of HCC cells (~85% and 90% cell death in Huh7 and Hepa1–6 cells, respectively). NPs achieve mannose-mediated anti-proliferation and anti-metastasis effects in HCC cells	Plumbagin 1 mg/kg and dihydrotanshinone I 3 mg/kg, every 2 d/7x	IV	Antitumor efficacy was (*p* < 0.001) enhanced. A long-term survival of animals was achieved	None	[50]
	All-trans retinoic acid	Doxorubicin	Micelle	Active targeting (hyaluronic acid)	ND	lower migration rate and greater inhibitory effect	Doxorubicin 3 mg/kg and all-trans retinoic acid 2.2 mg/kg, every 2 d/7x.	IV	Inhibition of 85.8% on tumor size. Decreased lung nodules.	Not reported	[51]
Lung	Irinotecan	Doxorubicin	Micelle	Passive targeting ROS-responsive release	<1 at 0.58 ug/mL of doxorubicin and 5.77 of irinotecan in LLC	Enhanced cell proliferation inhibition rate	Doxorubicin 5.0 mg/kg and irinotecan 50.0 mg/kg, 3x (d 1, 4 and 9)	IV	Almost complete retardation of tumor growth during the 16-day observation period	Not reported	[52]
	Docetaxel	Cisplatin	Nano sphere	Passive targeting	ND	Enhancement of cytotoxicity	Docetaxel 3.8 mg/kg and cisplatin 2.1 mg/kg, every 4 d/15 d	IV	Outperformed all other treatment arms by blunting tumor growth the most in both lung cancer models	Low hepatic and nephrotoxicity and weight fluctuance	[53]
	Paclitaxel	Carboplatin	Nano sphere	Passive targeting pH-sensitive release	ND	Better anti-tumor effect	Paclitaxel 1 mg/kg and carboplatin 2 mg/kg, every 2 d/7x	IV	Tumor reduction index of 79%	Not reported	[54]
	Paclitaxel	Survivin siRNA	Nano sphere	Passive targeting pH-sensitive release	<1 at paclitaxel:siRNA 1:10	A greater anti-proliferative effect on A549 lung cancer cells than either treatment alone	Paclitaxel 7.5 mg/kg and survivin siRNA 3 mg/kg, every 3 d/4x	IV	Much more effective at shrinking the tumors than controls	Low systemic toxicity	[23]
	Paclitaxel	Doxorubicin	Micelles	Active targeting (Folic acid) pH-responsive release	<1	Efficient endocytosis	Every 3–4 d/number of d not clear	Intra tumoral	Lowest tumor weight and highest tumor inhibitory rate of 83%	Small weight loss	[55]
	Silybin	Paclitaxel	Micelles	Passive targeting	<1 at different proportions	Notable anti-angiogenesis effect with the tubular formation rate decreased to 60%	Silybin 10 mg/kg and paclitaxel 7 mg/kg, every 2 days/6x	IV	Promoted tumor vascular normalization, depleting TAFs and collagen, and ultimately improved penetration of NPs into tumor within A549 xenograft model.	No weight change	[56]
	Etoposide	Cisplatin	Nano sphere	Passive targeting	<1 at etoposide: cisplatin 1:1.8	Modestly better than free drugs	Etoposide 1.25 mg/kg and cisplatin 2.25 mg/kg 1x	IV	Significant tumor growth reductions	Low systemic toxicity was observed	[32]
	Paclitaxel	Cisplatin	Nano sphere	Passive targeting	ND	Further reduced non-small cell lung cancer cells survival	Cisplatin 3.9 mg/kg and paclitaxel 5.0 mg/kg + 3 × 5 Gy X-ray irradiation at 3 h, 24 h and 48 h after administration	IV	Statistically significant reduction in tumor growth compared to controls	Not reported	[57]
	Etoposide	Cisplatin	Micelles	Passive targeting	<1 (Cisplatin:etoposide:PM 4:8:10)	Uptake of both drugs is enhanced	Cisplatin 15 mg/kg and etoposide 30 mg/kg each 4 d/4x	IV	Most pronounced antitumor effect and decreased the final tumor volume to only ca. 350 mm³ and greater survival length	Not reported	[58]
Lymphoma	Vincristine	Quercetin	Lipid-polymeric NP	Passive targeting	<1 to Vincristine to Quercetin 2:1, 1:1 and 1:2	Superior in terms of cytotoxic activity as compared to their solution counterparts	20 mg/kg each every 7 d/number of d not clear	IV	Significant inhibition of tumor growth	Not reported	[59]
Ovarian	Adavosertib	Olaparib	Nano capsule	Active targeting (TMTP1 peptide)	<1 to 1:5	Synergistic effects in eliminating ovarian cancer cells	Adavosertib 5 mg/kg and olaparib 25 mg/kg, every 2 d/6x	IV	Tumor inhibition reached 93.5%	None	[60]
	Rhein	Doxorubicin	Micelles	Passive targeting	ND	Led to the arrest of SKOV3/DOX cells at the G0/G1 phase of cell cycle, accompanied by a decrease in cells in S and G2/M phase. And induced the highest apoptotic proportion.	Doxorubicin 5 mg/kg and rhein 7.5 mg/kg not clear frequency of administration	IV	Stronger anti-tumor role in SKOV3-bearing tumor samples. Tumor volume and weight were obviously smaller than controls	Not reported	[61]
	Wortmannin	Cisplatin	Nano sphere	Passive targeting	<1	Strongly and synergistically enhanced the drugs’ efficacy	Wortmannin 0.15 mg/kg and cisplatin 0.3 mg/kg 1x	IV	Reduced tumor growth rates significantly versus control (both *p* < 0.001)	Low off-target kidney and liver cytotoxicity	[31]
	Doxorubicin	Verapamil	mPEG-PLA nanoparticles	Passive targeting	ND	Increased intracellular concentration of DOX in A2780/DOX^R^ and SKOV3/DOX^R^ cells and a significant reduction in cell viability (enhanced apoptosis).	Doxorubicin 5 mg/kg and Verapamil 1 mg/kg 2 d/3x	IV	enhanced tumor suppression effects in nude mice bearing DOX-resistant tumors and prolonged survival time	Reversal of body weight loss induced by non-encapsulated drugs. Reduced the side effects on the liver and kidney functions	[33]

NP: nanoparticle; ND: not determined; w/: with; Adm: Administration; d: day(s); w: week(s); n°d/n°x: number of days per number of times; IV: Intravenous; IP: intraperitoneal.

## Data Availability

No new data were created or analyzed in this study. Data sharing is not applicable to this article.
